# 4505 Implementation of Real-World Data and Real-World Evidence in Clinical Studies

**DOI:** 10.1017/cts.2020.347

**Published:** 2020-07-29

**Authors:** Jessica Pham, Eunjoo Pacifici

**Affiliations:** 1 University of Southern California

## Abstract

OBJECTIVES/GOALS: Real-world studies have been gaining momentum in providing evidence of treatment effectiveness and hold great potential for facilitating the drug regulatory process. The U.S. Food and Drug Administration (FDA) has recognized this by providing a framework for using real-world data (RWD) to generate real-world evidence (RWE). The objective of this study is to assess the current level of RWE implementation in clinical studies. METHODS/STUDY POPULATION: Using keywords relevant to RWE, we reviewed studies on drugs, biologics, and medical devices published on PubMed in 2018. Information regarding the therapeutic area of focus, intervention type, study design, primary outcome, and data source was recorded. Further analyses of the three main therapeutic areas of study (oncology, cardiology, and infectious diseases) were performed to determine how RWE was being utilized. In addition, a broad “real-world” search was performed on Clinicaltrials.gov, from which we extracted relevant observational and Phase I, II, II/III, III, III/IV, and IV studies. A supplemental PubMed search was used to evaluate published studies in order to identify which field these trials were concentrated in and the outcome of interest.” RESULTS/ANTICIPATED RESULTS: After application of “real-world” search terms to PubMed, 995 hits were generated and of these, 311 studies were excluded. More than half of the studies were observational and retrospective in nature (64%) with 70% examining drug/biologic outcomes. RWE data sources were largely dominated by medical records and claims data. The primary uses of RWE across oncology, cardiology, and infectious diseases included supporting drug product effectiveness, assessing safety, and evaluating treatment patterns. Of the 207 RWE studies identified on ClinicalTrials.gov, 66 were cancer randomized controlled trials (RCTs), a majority of which were used for post-marketing safety evaluations. Further research will be conducted to determine the precise role of RWE in all studies (e.g. historical comparator, label expansion). DISCUSSION/SIGNIFICANCE OF IMPACT: By examining the use of RWE in regulatory decision making, we can inform stakeholders of the extent to which robust RWE studies complement evidence generated by RCTs. Thought to reflect a product’s performance in a broader and more diverse population, RWE can provide greater insight to clinical trial conduct and ultimately transform patient outcomes.

